# Can patients improve the quality of care they receive? Experimental evidence from Senegal

**DOI:** 10.1016/j.worlddev.2021.105740

**Published:** 2022-02

**Authors:** Roxanne J. Kovacs, Mylene Lagarde, John Cairns

**Affiliations:** a Department of Economics and Centre for Health Governance, University of Gothenburg, Sweden; b London School of Economics and Political Science, Department of Health Policy, United Kingdom; c London School of Hygiene and Tropical Medicine, Faculty of Public Health and Policy, United Kingdom

**Keywords:** Quality of care, Field experiment, Patient behaviour, Provider behaviour, Patient-provider interaction, Standardised patients, Communication, Senegal

## Abstract

Providers in many low and middle-income countries (LMICs) often fail to correctly diagnose and treat their patients, even though they have the clinical knowledge to do so. Against the backdrop of many failed attempts to increase provider effort, this study examines whether quality of care can be improved by encouraging patients to be more active during consultations. We design a simple experiment with undercover standardised patients who randomly vary how much information they disclose about their symptoms. We find that providers are 27% more likely to correctly manage a patient who volunteers several key symptoms of their condition at the start of the consultation, compared to a typical patient who shares less information. Lower performance in the control group is not due to providers’ lack of knowledge, an incapacity to ask the right questions, or a response to time or resource constraints. Instead, providers’ low motivation seems to limit their ability to adapt their effort to patients’ inputs in the consultation. Our findings provide proof-of-concept evidence that interventions making patients more active in their consultations could significantly improve the quality of care in LMICs.

## Highlights

• We use standardised patients to test if quality of care increases when patients share more information in rural Senegal
• Providers are 27% more likely to correctly manage patients who volunteer more information at the start of the consultation
• Low performance when patients share less information is not due to providers’ lack of knowledge or clinical skills
• Instead, low motivation may limit providers’ ability to adapt their effort to patients’ inputs in the consultation.
• Encouraging patients to be more active in consultations could improve the quality of healthcare in low-income settings.

## 1 Introduction

Healthcare providers in low and middle-income countries (LMICs) often fail to diagnose common and life-threatening medical conditions, and provide incorrect treatments to their patients (Daniels et al., 2017, Das et al., 2016, Das et al., 2016, Mohanan et al., 2015, Sylvia et al., 2015). This is not necessarily because they lack appropriate knowledge; studies from India (Mohanan et al., 2015), China (Sylvia et al., 2017) and Rwanda (Gertler & Vermeersch, 2013) show that providers often failed to treat patients appropriately even though they were able to correctly identify and manage their illness in a knowledge test. The same mismatch between actual and best-possible practice was found in Tanzania when doctors were observed by a peer (Leonard & Masatu, 2010b). These “know-do gaps” suggest that poor quality care in LMICs is at least partially due to providers not investing enough effort during consultations.

Many previously tested interventions to improve the quality of care have centred on healthcare providers. Early interventions focused on providers’ working environment, for example, by reducing shortages in essential drugs and equipment (Akin et al., 1995, Hotchkiss, 1993), or improving supervision and managerial support (Dieleman et al., 2009, Ross-Degnan et al., 1997). Training workshops to improve providers’ clinical knowledge have also been implemented in countless settings (Althabe et al., 2008, Wong et al., 2015). More recently, many governments pinned their hopes in financial incentives to improve provider performance (Gertler et al., 2011, Witter et al., 2012). Unfortunately, these programmes have been largely unsuccessful at significantly improving the quality of healthcare in LMICs (Diaconu et al., 2021, Eijkenaar et al., 2013, Rowe et al., 2005). In this paper we explore a new avenue for improving the quality of medical care in LMICs, focusing instead on the role of the patient during the consultation.

We propose to think about the quality of care in a clinical consultation as the output of a simple production function with two inputs: provider effort and patient effort. For providers, ‘effort’ refers to actions undertaken to establish the patient’s diagnosis: asking questions to elicit relevant symptoms and information about the patient’s medical history and conducting physical examinations as necessary. Evidence from LMICs suggests that provider effort can be very limited, with providers in some settings asking only two questions on average in a consultation and undertaking no physical examination (Das et al., 2012). For patients, ‘effort’ consists of volunteering information about their symptoms or concerns, and responding as best as possible to providers’ questions. It follows from this framework that improving the output of a clinical encounter can be done by increasing either type of effort, not solely by seeking to increase providers’ input. Building on a large body of work from medical anthropology, patients’ ability to present their concerns or divulge symptoms has long been recognised as a key determinant of the quality of care (Heritage & Maynard, 2006). Yet, paradoxically, interventions in high-income countries seeking to increase patients’ disclosure of information have almost exclusively targeted providers and their communication skills (Kelley et al., 2014). Only recently have awareness campaigns started to target patients themselves, encouraging them to take a more active role. For instance, a campaign by the Centres for Disease Control in the United States encouraged patients to reduce the risk of a missed sepsis diagnosis, by directly asking their doctor “*Could this be sepsis*?” (CDC, 2017). Similarly, when providing medical information to the public, the Mayo Clinic encourages patient involvement by advising them to “*make a list of any symptoms*” before the consultation and disclosing them to their doctor (Mayo Clinic, 2020a, Clinic, 2020b).

Against the backdrop of many failed attempts to improve provider effort in LMICs, this paper presents a proof-of-concept study that demonstrates the potential role of patients in improving outcomes of clinical encounters. Specifically, we provide novel causal evidence of the positive impact achieved when patients volunteer more symptoms at the outset of a consultation, instead of waiting for providers to ask specific questions. The study consists of a simple experiment involving undercover standardised patients (SPs) in Senegal. SPs are healthy individuals trained to attend medical consultations, report specific symptoms to providers and subsequently record what happened during the consultation. The SPs presented a classic case of pulmonary tuberculosis (TB) in 197 public-sector primary care facilities in rural areas. Facilities were randomly assigned to receive a SP following one of two scenarios. In a control scenario reflecting the usual subdued attitude of patients in the study setting, SPs volunteered only one main symptom at the start of the consultation – requiring the provider to interrogate the patient to retrieve other relevant information and exclude alternative diagnoses. In the other scenario, the patient volunteered three key symptoms of TB – potentially allowing providers to infer the correct diagnosis without the need for much more information.

Our results indicate that this simple change in communication has large positive effects on quality of care received. Providers are 27% more likely to manage correctly a patient who volunteers several key symptoms of their condition at the start of the consultation, compared to a patient who requires a more thorough interrogation. This effect is much larger than that of previous interventions focused on improving provider effort. Our findings also provide insights into the production function of quality of care in our setting. We find that providers do not respond sufficiently to patients’ inputs, as their effort is largely independent of patient symptom disclosure. If providers adapted their effort to the level or quality of information disclosed initially by patients, they should seek more information from patients who initially divulge fewer symptoms. Yet, this is not the case: providers exert largely the same effort regardless of the amount of information volunteered by the patient. Finally, we explore some potential mechanisms behind our results, which point to the role of low provider effort in retrieving relevant information from the patient. By showing that providers ask about the undisclosed symptoms in a knowledge test but not in the actual consultation, we rule out a lack of knowledge as a reason for low performance in the control group. We also exclude the time or health system resource constraints faced by providers as a factor limiting their performance. Finally, we find suggestive evidence that low motivation explains why providers with less information fail to invest the necessary effort in the clinical encounter. It is possible that, instead of adapting their effort to the patient case, demotivated providers apply a simple heuristic (mental shortcut), and classify patients as having a mild or severe illness based solely on the number of symptoms volunteered.

This study contributes to three strands of literature. First, this paper contributes to the growing body of empirical studies exploring how to improve the low quality of care in LMICs. Most of the literature (and policy debates) has focused on interventions targeting providers, with a large body of recent work in LMICs studying the effects of financial incentives to increase provider effort (Diaconu et al., 2021). Other studies have highlighted the potential role for communities and patients to increase providers’ effort through accountability mechanisms outside the consultation room (Björkman and Svensson, 2009, Nyqvist et al., 2017). To our knowledge, only two other studies have explored the causal effect of patients’ disclosure of information on the quality of care in LMICs, also by using SPs. In China, Currie et al. (2011) showed that patients with flu-like symptoms who display knowledge of appropriate antibiotics are less likely to receive unnecessary antibiotics. In a study with SPs portraying a case of TB in India, providers were more likely to correctly manage patients who presented more information about their case, in the form of results of past medical exams (Kwan et al., 2018). Unlike these two studies, ours demonstrates the potential power of a simpler mechanism, plausibly costless to the patient. Unlike the study in India where patients’ information derived from costly actions undertaken previously (i.e. past treatments or a chest x-ray), we show that quality of care increases simply when patients share more information at the outset. This does not require the more sophisticated knowledge of the patient used by Currie et al. (2011), which can be hard to achieve. Instead, our study provides rigorous evidence that a simple change in patient communication at the start of the consultation could have large impacts. Although the responsibility for receiving high-quality care should not fall on patients, healthcare markets in many LMICs currently do not give providers an incentive to exert high effort, and government interventions have so far been mostly ineffective. Policymakers might therefore want to test interventions that encourage patients to play a more active role in the consultation.

Second, we contribute to the literature on communication in the patient-clinician relationship. Starting with ethnographic studies in medical anthropology, there is a long tradition in the health literature to explore the nature of the communication between patients and doctors, and their power dynamics (Heritage and Maynard, 2006, Timmermans, 2020). A lot of this work takes place in high-income settings and draws on observational studies, many qualitative (Ridd et al., 2009), which limit the ability to identify the causal role of communication on health-related outcomes. Existing experimental studies have looked at interventions improving doctors’ communication skills (Harrington et al., 2004, Kelley et al., 2014), although there has been an increased interest in interventions improving patients’ ability to communicate (D’Agostino et al., 2017). We add to this body of work in several ways. The experiment we present specifically tests whether patients’ improved presentation of their symptoms can influence the outcome of medical consultations – a notion that is generally accepted but, to our knowledge, has never been tested directly (Heritage and Maynard, 2006, Timmermans, 2020). Next, our main outcome of interest is an objective measure of providers’ clinical decision-making and the quality of care provided in the clinical interaction. Our study stands in contrast to a literature that has mostly focused on patients’ reported outcomes – their satisfaction, adherence to treatment, information recall and health care visits (Harrington et al., 2004, Kelley et al., 2014). Finally, our study is set in a Sub-Saharan African country, where most previous work on communication between patients and providers has been qualitative (Kwame and Petrucka, 2020, Long et al., 2008).

Lastly, we contribute to the literature exploring provider behaviour in LMICs and what contributes to their poor performance. A body of qualitative and mixed-methods research examining the patient-provider interaction has highlighted a number of inadequate provider behaviours that are detrimental to the provision of high-quality care and future care seeking, from rudeness and verbal abuse to patient neglect and physical mistreatment (Campero et al., 1998, Grossmann-Kendall et al., 2001, Mannava et al., 2015, Silal et al., 2012). This literature has often blamed these issues on excessive workload and shortages of staff (Kwame & Petrucka, 2020), which do not explain providers’ failure in many settings (Das et al., 2018), including the one we study. Following a growing body of quantitative studies (Gertler and Vermeersch, 2013, Leonard and Masatu, 2010b, Leonard and Masatu, 2010a, Mohanan et al., 2015, Sylvia et al., 2017), we show significant know-do gaps, between what providers can do in theory and what they do in practice, suggesting that low provider effort is a significant barrier to higher quality care. We further unpack the “black box” of provider effort, showing that providers’ inability to take patients’ history may drive misdiagnoses and poor quality of care. Our results reconcile the quantitative and qualitative strands of literature by highlighting the role of providers’ poor communication skills, and providing quantitative estimates of its negative impact on the quality of care received by patients.

## 2 Background

### 2.1 Primary care in Senegal

This study was undertaken in Senegal, where the quality of healthcare has been shown to be relatively poor. Previous work suggests that public healthcare workers have a low level of clinical knowledge, as only 4% are able to correctly diagnose a case of severe malaria and only 33% are able to correctly diagnose a case of diarrhoea with dehydration (WB, 2012). In addition, providers appear to invest low levels of effort, completing only 60% of recommended actions in ante-natal consultations and 43% in paediatric consultations (Kruk et al., 2017). This suggests that levels of process quality of care are lower in Senegal than in similar countries in sub-Saharan Africa (Kruk et al., 2017).

This research took place in public primary care facilities in four (of fourteen) regions (Ziguinchor, Sédhiou, Tambacounda, Kédougou), home to 13% of the Senegalese population (ANSD, 2014) – see Fig. A1 in the Appendix A1. These rural areas are amongst the most disadvantaged of the country. Most households living in these areas (68%) are in the two poorest wealth quintiles in the country and engage in subsistence agriculture (DHS, 2015). Use of healthcare services is limited, with only about 65% of households seeking any form of healthcare when their child had their last episode of fever, cough or diarrhoea (DHS, 2015). Since there are hardly any private provider in rural Senegal, public primary healthcare facilities (health posts) are the main point of access for patients who seek care. They are typically staffed by a nurse and a midwife, as well as unskilled health workers with paramedical or no medical training. If all providers are present at the facility, nurses and midwives conduct preventative and curative primary care consultations and unskilled providers take on supporting roles. When nurses and midwives are absent, unskilled health workers deliver services.

### 2.2 Tuberculosis in Senegal

TB is an important public health concern in Senegal. The Ministry of Health identifies TB as one of its top priorities and spends approximately 11 million USD (3.6% of total health expenditure) on its national TB programme each year (WHO, 2015). The estimated incidence rate of TB in Senegal is considerably lower than in other countries in the region with a higher burden of HIV/AIDS. Modelled estimates suggest that 21,000 people in Senegal are living with TB (139 per 100,000 population) (WHO, 2015) – which is comparable to South Sudan, Vietnam or Chad (WB, 2019). As is the case in many countries, TB is more prominent in urban areas. One study reports TB incidence rates in the capital Dakar as more than twice those in rural areas (Diop et al., 2002). However, 87% of facilities in the study areas are able to provide first-line treatment for TB (DHS, 2016).

Pulmonary TB was chosen as the focus of this study because it is a serious condition, for which widespread information and medical protocols exist. In addition, it generally manifests itself through several clearly identifiable symptoms including a persistent cough, unexplained weight loss, night sweats, and blood in the sputum. Patients often also experience fatigue, fever, chills or loss of appetite. As in many other LMICs, clinical guidelines in Senegal indicate that providers should screen for TB a patient reporting a cough that has lasted for over two weeks. The most common form of TB screening used in Senegal is a sputum test, although other accepted approaches include a Mantoux test (tuberculin skin test) or doing a chest X-ray. Importantly, clinical guidelines require providers to confirm that a patient is infected with TB before prescribing medication. There is little evidence on the quality of care provided to patients with symptoms of TB, although one study found that patients frequently receive inadequate combinations or dosage of TB medication (Diop et al., 2002).

## 3 Experimental design

### 3.1 The standardised patient case

SPs are healthy individuals trained to visit providers, report specific symptoms, answer questions based on a pre-defined script and subsequently record what happened during the consultation. SPs usually complete a standardised checklist providing details on the questions asked, examinations performed, recommendations made, and drugs prescribed during the consultation. Among existing methods for measuring quality of care (such as interviews with patients, clinical record audits or observations of clinical consultations), SPs are often regarded as the gold standard (Kwan et al., 2019).

In collaboration with local and international health professionals, and drawing on previous studies (Das et al., 2015), we developed a ‘textbook’ case of a patient with symptoms of pulmonary TB. To play the SPs, we recruited eight enumerators, all men in their early thirties from the study areas who had passed a medical screening showing they were healthy. To portray the role of someone who would have lost weight and look tired, we chose very slim or slightly underweight men who did not have a ‘healthy glow’. SPs underwent a medical check-up and were trained for two weeks to rehearse a detailed script containing information on the SP’s personal and medical history as well as answers to an extensive list of questions that providers might ask during the consultation (see Appendix A2). SPs did not fake coughing during the consultation because of the challenges to mimic a productive cough with consistency and credibility. As part of their background story, SPs indicated that they were living and working on a construction site in Dakar but at the time visiting a relative in the area. We also provided information and names of local leaders and residents in the area, that SPs could use if they needed to provide more details of their whereabouts and ‘alibi’.

### 3.2 Experimental design

**Fig. 1:**
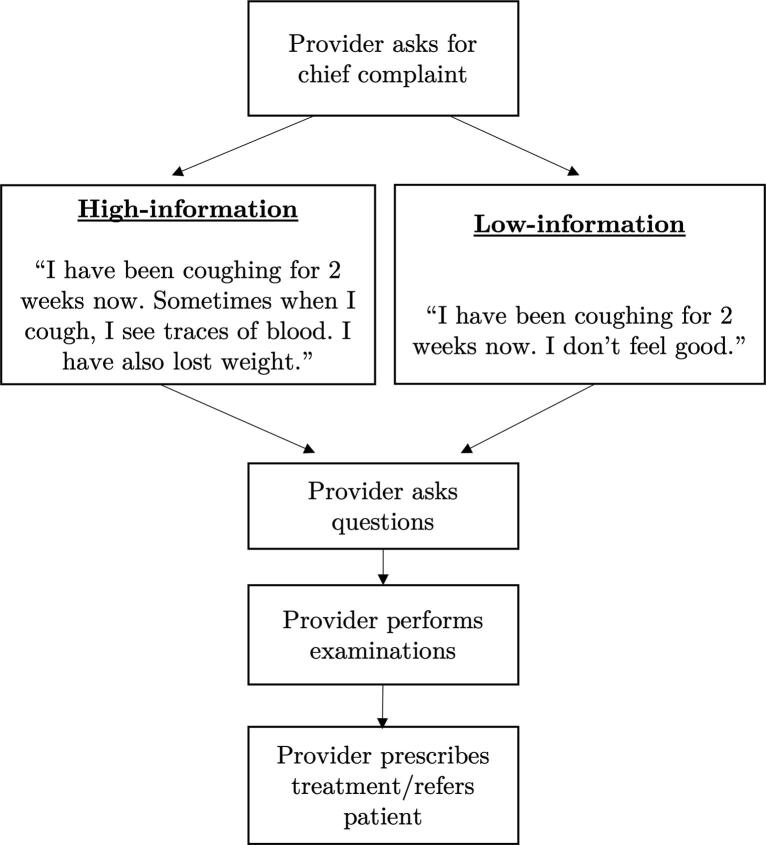
Experimental design.

In the experiment we randomly vary the amount of information disclosed by patients at the start of the consultation – see Fig. 1 for an overview of the study design. Specifically, for any given visit, each SP used one of two opening statements to describe their chief complaint:

- a generic introductory statement (low-information/control group): “*I have been coughing for two weeks now. I don’t feel good*”;
- or a detailed one (high-information/treatment group): “*I have been coughing for two weeks now. Sometimes when I cough, I see traces of blood. I have also lost weight.*”

To avoid confounding the treatment effect with an individual SP effect, the statements were randomised at the SP level. Before each facility visit, field supervisors verified that SPs knew which opening statement had to be used that day. After each consultation, SPs were asked which opening statement they had used, to record possible non-compliance (which occurred twice). Overall, the low-information introduction was used in 49% of consultations (n = 97) and the high-information introduction in 51% (n = 100) of consultations.

While the information *initially* volunteered by patients varied between the two groups, all SPs were trained to respond in the same way to providers’ questioning. SPs were trained to report other symptoms or details in their medical history if, and only if, they were asked specifically about them. Specifically, *if asked* by the provider, patients in the low-information group reported that they had been coughing blood or losing weight. SPs were left with little room to improvise when open questions, such as “*Do you have any other symptoms?*” were asked. All SPs were trained to respond to such questions by saying “*I feel very tired and exhausted*” – to avoid unobserved variation in the information revealed to providers.

Patients in the low-information group report a persistent cough of more than two weeks, the most typical symptom for TB. Although according to national guidelines this is a sufficient symptom to screen the patient for TB, other diseases could explain the persistent cough (pneumonia, bronchitis or even asthma). Providers would need to ask further questions to confirm the suspicion of TB with other more specific symptoms or rule out these alternative conditions. Patients in the high-information group volunteer three symptoms: persistent cough, blood in sputum and recent weight loss. Together they form a highly characteristic picture of TB, making it at the outset the most likely diagnosis.

The opening statement in the low-information group was designed in collaboration with local healthcare providers, to represent patients’ attitude and what they might typically share at the start of a consultation. Hence it is a suitable benchmark to test the effect of disclosing additional information on the quality of care and provider behaviour.

## 4 Data

### 4.1 Consultation data and main outcomes

Within an hour of their visit, SPs were debriefed with a standardised questionnaire to collect information on the questions asked, physical examinations performed, recommendations made, and drugs and tests given or prescribed (see Appendix A2).

We phoned all providers approximately eight weeks after SP visits to verify whether they had suspected any patients to be a fake SP during the consultation. In total, 17 providers reported some suspicions (8.6% of consultations), eight in the low-information group and nine in the high-information group. This detection rate is comparable to other studies conducted in LMICs and relatively low considering that the study was done in a rural area (such as, Das et al., 2012, Sylvia et al., 2015).

All providers who took part in the study gave their written consent to receive SPs. Providers were told during prospective facility visits that SP visits would take place within the next 6–8 months – without specifying the clinical case that would be portrayed by SPs. For all providers who were absent or not yet in post when facility visits took place (*n* = 63 providers or 32%), we obtained consent to use data retrospectively.

The main outcome of interest by which we measure quality of care is a binary measure of correct case management, which indicates whether the recommendations and treatments given to the SP were clinically indicated. Local experts determined what constitutes correct management based on national TB treatment guidelines. Specifically, providers who referred SPs to a higher-level facility with capacity to conduct TB screening or ordered any form of TB screening – either specific to TB (sputum test or Mantoux test) or not (chest x-ray) – were deemed to have managed the case correctly.

The second outcome of interest captures the effort exerted by providers during the consultation, captured by two similar measures. First, we computed the proportion of relevant history questions asked and physical examinations performed (Appendix A3). Second, following Das & Hammer (2005) our preferred measure of effort is a latent score of effort computed following Item Response Theory (IRT), which allows to discriminate better amongst providers by attributing greater weighs to items that are more difficult (see more details in Appendix A3). The proportion of relevant history and the IRT score were computed separately for the two groups as providers faced different patient inputs, requiring them to retrieve different elements of information.^1^

The third and final measure of effort is the duration of the consultation. We use the start and end time of the consultation, as recorded by the SPs.

### 4.2 Facility and provider data

SPs visited a random sample of 197 public primary care facilities between November and December 2016, representing 83% of the 238 primary care facilities in the study regions. Approximately six months before the SP visits, all facilities were visited. A questionnaire was administered to the person in charge of the facility to gather information on the facility characteristics and providers’ working environment – including availability of essential drugs, basic equipment and clinical guidelines (Appendix A4). All providers present in the facility at that time completed a questionnaire on their socio-economic characteristics, educational background, employment history, and intention to leave the facility. We use the latter as a proxy for their motivation.

All healthcare workers who indicated conducting curative adult consultations were asked to complete an additional task, to collect information on their clinical knowledge. Each provider completed five clinical vignettes, involving diagnosis of hypothetical patients presented through role-play by a trained enumerator (as in Leonard et al., 2007, Mohanan et al., 2015). As described in detail elsewhere (Kovacs et al., 2020), hypothetical patients in these vignettes presented cases of dysentery, malaria, pneumonia, asthma and stable angina. The hypothetical patient started the ‘consultation’ by describing their main symptom. Providers could then obtain more information by questioning the ‘patient’ and could indicate which physical examinations or laboratory tests they would perform. At the end of the exercise, providers indicated how they would treat the ‘patient’. All actions taken by the provider during the role-play (history questions asked, physical examinations and test results requested, advice given, and treatments recommended) were recorded by another enumerator.

Providers also assessed a hypothetical patient with TB symptoms, which had the same medical history as the SP case and responded in the same way to providers’ questioning. Based on a random draw, the hypothetical patient used either the generic introduction, “*I have been coughing for two weeks now. I don’t feel good*”, to present their main symptom (low-information vignette) or the more detailed introduction, “*I have been coughing for two weeks now. Sometimes when I cough, I see traces of blood. I have also lost weight”* (high-information vignette). We use data from clinical vignettes to capture whether providers know how to correctly manage patients with TB and whether they know which questions should be asked and which physical examinations should be performed. In a similar way to what we do with data from SPs, we also construct a clinical competence index using IRT which captures a provider’s ability to complete relevant actions (history questions, physical examinations) during the role play.

We were unable to match all of the SP visits with individual provider data. First, *n* = 63 who saw SPs were not present at the facility at the time of the initial survey – either because they were absent or had not been posted to the facility yet. While we have no data on clinical knowledge for these providers, we still managed to obtain basic information for all but six of them. Second, *n* = 31 providers received an SP even though they reported not conducting curative adult consultations during the facility visit, and had therefore been excluded from the vignette survey.

### 4.3 Balance checks

Table 1 presents the characteristics of the sample and checks that the two groups of consultations are balanced on provider and facility characteristics.

**Table 1:** Balance checks.

		Treatment		Control		p-val.
		Mean	SD	Mean	SD	
**Panel A: Facility characteristics (N = 197)**						
Health post		0.90	0.30	0.96	0.20	0.11
Target population size (thousands)		7.60	6.76	7.28	5.70	0.72
Competition (facilities in 5 km radius)		1.21	1.73	1.69	3.24	0.19
Distance to next higher-level facility (km)		35.23	32.95	38.11	37.35	0.57
% of essential drugs and equipment available		0.79	0.08	0.78	0.09	0.37
Treatment guidelines for TB available		0.43	0.50	0.46	0.50	0.63
Consultation volumes on average day		8.69	8.34	8.89	7.66	0.86
Patients waiting when SP arrived		4.49	5.65	5.33	5.81	0.30
**Panel B: Provider characteristics (N = 191)**						
Male		0.57	0.50	0.62	0.49	0.48
Skilled (nurse, doctor, midwife)		0.51	0.50	0.45	0.50	0.42
Work experience (years)		9.91	8.68	10.45	9.69	0.68
Undertook training on TB		0.50	0.50	0.47	0.50	0.72
Intends to quit job (N = 134)		0.29	0.46	0.25	0.44	0.62
Provider TB knowledge (N = 119)						
Correctly managed TB vignette		0.80	0.41	0.82	0.39	0.78
Competence index for TB (IRT score)		−0.09	1.03	0.09	0.97	0.34
Provider clinical knowledge (N = 119)						
Correctly managed dysentery vignette^ᴥ^		0.33	0.47	0.43	0.50	0.24
Correctly managed malaria vignette		0.24	0.43	0.17	0.38	0.34
Correctly managed pneumonia vignette^ᴥ^		0.91	0.28	0.90	0.30	0.80
Correctly managed asthma vignette		0.58	0.50	0.50	0.50	0.41
Correctly managed angina vignette		0.64	0.48	0.68	0.47	0.65

Note: p-values of t-tests for means and chi-squared tests for proportions. ^ᴥ^For the dysentery and pneumonia vignette N = 118.

As shown in Panel A, information on facility characteristics is available for all 197 consultations. 93% of consultations occurred in health posts, rather than in larger and better equipped health centres. Facilities serve an average population of 7,400 and are located in remote areas, on average 37 km away from the nearest higher-level institution. Nonetheless, facilities were relatively well-resourced at the time the facility survey was conducted (i.e. six months before SP visits), with 78% of essential drugs and equipment available – which is comparable to a recent national assessment of service readiness (DHS, 2017). Treatment guidelines for TB were available in roughly half of the facilities. Consultation volumes are low in the study facilities; based on data from clinical records and the number of providers present on an average day, providers conduct nine consultations per day on average. When SPs arrived at health facilities, there were on average five other patients waiting to be seen. Based on our data, consultations for TB last 11 min on average. This implies that providers spend less than two hours a day consulting patients. Whilst providers have other responsibilities (administrative work, community outreach) this suggests that they are unlikely to face important time constraints when consulting patients.

As shown in Panel B, the two groups are also balanced on all observed provider characteristics. Providers have ten years work experience on average, 48% have a professional qualification (doctor, nurse, midwife), and 51% had attended a training workshop on TB management in the past two years. For the *n* = 134 providers who were asked about their intention to leave, 27% indicated that they wanted to quit their job and are therefore categorised as having a “low motivation”. Of the *n* = 119 providers who completed the clinical vignettes, 74% know how to manage a case of TB correctly – compared to 38% for dysentery, 20% for malaria, 90% for pneumonia, 54% for asthma and 66% for angina. Providers completed on average a third of the relevant actions (history questions, physical examinations) in the TB vignette.

### 4.4 Econometric approach

Because of the randomised design, we can identify the effect of patients volunteering additional information by simply estimating the following OLS regression:

$$Y_{ispf}=\beta_{0}+\beta_{1}{\mathrm{Disclose}}_{i}+\beta_{2}Z_{f}+\beta_{3}X_{p}+\delta_{s}+\varepsilon_{ispf}$$

where $Y_{ispf}$ is the outcome of interest for consultation i between SP s and provider p in facility f . ${\mathrm{Disclose}}_{i}$ is a binary variable equal to 1 if the SP used the high information opening statement. The coefficient $\beta_{1}$ can be interpreted as the effect of patients volunteering more relevant information on the quality of case management. The remaining variables control for other possible determinants of the quality of case management. $Z_{f}$ refers to a vector of facility characteristics and $X_{p}$ refers to a vector of provider characteristics, which are only available for a sub-sample of providers (as shown in Table 1). We also include enumerator fixed effects ($\delta_{s})$.

One of the identifying assumptions we are making is that unobserved effort from each SP is not systematically related to the information they provide in their opening statement. If SPs are putting more emphasis on their role when they are providing the high (low)-information introduction, this would imply that our estimates are a lower (upper) bound.

## 5 Results

### 5.1 Impact of patient’s information disclosure on quality of care

Overall, 68% of providers correctly managed the SP case: 60.9% (*n* = 120) ordered a sputum test, 0.5% (*n* = 1) a chest x-ray, and 6.6% (*n* = 13) referred the patient to a higher-level facility, without giving more specifics. Providers who managed SPs incorrectly most often simply prescribed antibiotics (89%), generally in combination with pain relief (such as paracetamol).

Looking at the two different types of patients, we find that 60% of SPs are correctly managed in the control group, compared to 76% of SPs in the treatment group (p < 0.05). Table 2 presents the results of several regressions formally estimating this difference and using a range of controls to increase the precision of the estimates. Column 1 presents the results of an unadjusted model, columns 2 and 3 presents the estimates with a full set of facility and healthcare worker controls, while in column 4 we use the lasso estimator to select the controls following the post-double-selection (PDS) methodology introduced in Belloni et al. (2013). All models show consistent results, suggesting an increase in the probability of correct management by about 16 percentage points, which corresponds to an increase of 27% in the probability of correct case management.

**Table 2:** Effect of information disclosure on correct case management.

	(1)	(2)	(3)	(4)
Patient discloses more information	0.162**	0.168***	0.164**	0.160***
	(0.066)	(0.064)	(0.064)	(0.062)
Mean (SD) in low-information group	0.60		0.60	
	(0.49)		(0.49)	
Facility characteristics	No	Yes	Yes	No^†^
Provider characteristics	No	No	Yes	Yes^†^
R-squared	0.030	0.176	0.208	–
Observations	197	197	191	191

Notes: Results from OLS regressions with robust standard errors are reported. Facility and provider characteristics are as shown in Table 1 (type of facility, target population, competition, distance to higher-level facility, facility participation in a results-based financing scheme, proportion of essential drugs and equipment available; provider gender, skill, experience, training). All models control for SP (enumerator) fixed effects. Model 2 and Model 3 control for the number of patients waiting when SPs arrived and for whether providers had been informed of SPs visits beforehand. ^†^ Model 4 includes only controls selected via post-double-selection (PDS) lasso (i.e. SP fixed effects as well as provider skills). *** p < 0.01, ** p < 0.05, * p < 0.1.

Overall, these results suggest that, everything else being equal, patients can affect the quality of care they receive by volunteering information about their symptoms.

As explained in section 4.1, not all management choices are necessarily specific to a diagnosis of TB. Hence, one concern could be that our results are driven by the non-specific diagnoses. This is not the case. Appendix Table A3 shows that when we use a more restrictive and TB-specific definition of correct management – i.e. ordering a sputum test – providing more information increases the probability of correct management by 14 percentage points, equivalent to the same 26% relative increase we found.

### 5.2 The role of provider effort

We now examine the mechanisms that may drive the better management of patients who volunteer more information. Ideally, whether a patient initially discloses several symptoms or not should not change the quality of care provided, as any information gap can be closed if providers question the patient to retrieve any relevant information. One way to test for the presence of such a “catch-up” effect is by exploring whether healthcare providers in the control group invest more effort than those in the treatment group, who receive more information on symptoms.

Table 3 presents the results from OLS regressions using three different measures of effort. Columns 1 and 2 present results for our preferred measure of effort, an index score of latent effort computed following IRT. In columns 3 and 4, we use the unweighted proportion of relevant items on the checklist completed by providers. Finally, in columns 5 and 6 effort is proxied by the duration of the consultation. In general, we cannot rule out the hypothesis that the effort exerted by providers under the two different conditions is the same. In five out of six specifications, we find no evidence of a difference. When controlling for facility and provider characteristics, there is some weak evidence suggesting that providers who hear less information complete 4% more history questions and physical examinations than those with more information (Column 4, p = 0.07).^2^ Overall, our results do not suggest that provider effort is responsive to the amount of information volunteered by patients.

**Table 3:** Effect of disclosing little information on provider effort.

	Effort (IRT score)		% questions asked and examinations done		Duration (minutes)	
	(1)	(2)	(3)	(4)	(5)	(6)
Patient discloses *less* information	0.050	0.075	0.033	0.038*	−0.192	−0.077
	(0.145)	(0.146)	(0.021)	(0.021)	(0.712)	(0.730)
Mean (SD) in high-information group	0.00	−0.02	0.33	0.32	11.07	11.12
	(1.06)	(1.06)	(0.15)	(0.15)	(5.52)	(5.58)
Facility characteristics	Yes	Yes	Yes	Yes	Yes	Yes
Provider characteristics	No	Yes	No	Yes	No	Yes
R-squared	0.151	0.189	0.137	0.159	0.260	0.317
Observations	197	191	197	191	197	191

Notes: Results from OLS regressions with robust standard errors are reported. Facility and provider characteristics are as shown in Table 1 (type of facility, target population, competition, distance to higher-level facility, facility participation in a results-based financing scheme, proportion of essential drugs and equipment available; provider gender, skill, experience, training). All models control for the number of patients in the waiting area when SPs arrived as well as SP fixed effects. The IRT score has a mean of zero and a standard deviation of one. *** p < 0.01, ** p < 0.05, * p < 0.1.

Notes: The probability of correct case management is plotted separately in the high-information group (left) where providers hear about the two key symptoms at the start of the consultation. The three bars on the right present separately the proportion of correct management for the three possible cases in the low-information group: providers ask no, one or two questions about the two key symptoms). Error bars show 95% confidence intervals.

A second way to investigate whether providers in the low-information group “catch-up” is by examining whether information about the (TB-specific) “missing” symptoms is asked about. Results indicate that when this key information is not volunteered by the patient, it is generally not asked about by providers. Only 8% of providers in the low-information group ask about the two additional pieces of information volunteered by patients in the high-information group (weight loss and blood in sputum) and 34% ask for information on one of these. Thus, in the majority (58%) of low-information consultations, providers request neither of the additional pieces of information volunteered by the treatment group.

**Fig. 2:**
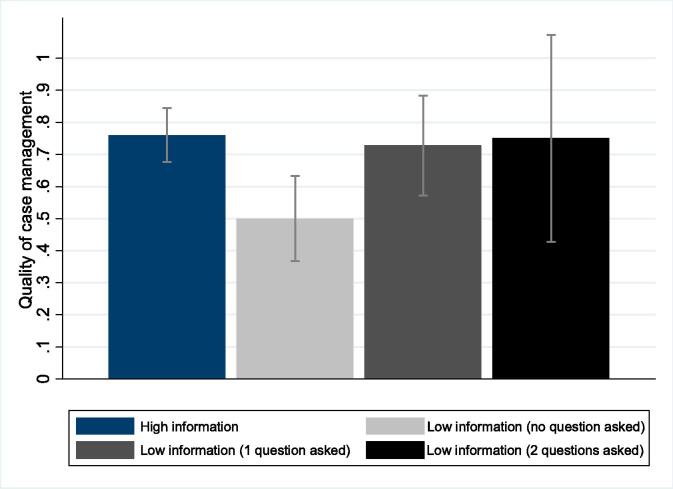
Probability of correct case management and information about key symptoms.

Going one step further, we contrast the proportion of correct case management in the high-information group, to the same outcome in the low-information group depending on the amount of information retrieved. Fig. 2 provides some illustrative evidence suggesting that the difference between the two groups is driven by providers not asking for either key symptom that is not initially disclosed by patients. We find that the predicted probability of correct case management does not differ between providers in the low-information group who collect one or both pieces of information and providers in the treatment group. Only providers who collect none of the additional information that is volunteered by the high-information group (i.e. the majority of providers) perform significantly worse. This result is confirmed formally in the regression results shown in Table 4. Providers in the low-information group who retrieve no information about the two symptoms are 26 percentage points (34%) less likely to manage patient cases correctly than those in the treatment group. This suggests that one of the reasons behind the poor quality of care is the inability to retrieve relevant information by interrogating the patient appropriately.

**Table 4:** Moderating effect of provider effort on the impact of patient’s limited information disclosure.

	(1)	(2)	(3)
Low information × no question asked	−0.260***	−0.254***	−0.250***
	(0.076)	(0.076)	(0.075)
Low information × 1 question asked	−0.033	−0.011	−0.024
	(0.092)	(0.093)	(0.085)
Low information × 2 questions asked	−0.010	−0.150	−0.078
	(0.168)	(0.172)	(0.161)
Mean (SD) in high-information group	0.76	0.77 (0.42)	
	(0.43)		
Facility characteristics	No	Yes	No^†^
Provider characteristics	No	Yes	Yes^†^
R-squared	0.060	0.235	–
Observations	197	191	191

Notes: Results from OLS regressions with robust standard errors are reported. Providers in the high-information group are the reference category. Facility and provider characteristics are as shown in Table 1 (type of facility, target population, competition, distance to higher-level facility, facility participation in a results-based financing scheme, proportion of essential drugs and equipment available; provider gender, skill, experience, training). All models control for SP fixed effects. ^†^ Model 4 includes only controls selected via post-double-selection (PDS) lasso (i.e. SP fixed effects as well as provider skills). *** p < 0.01, ** p < 0.05, * p < 0.1

Appendix Tables A5 and A6 show the full list of questions asked and physical examinations done by providers in the two groups. We find that providers in the high-information group were significantly more likely to ask whether the patient had blood in his sputum (perhaps as a confirmatory question) and whether people around them were coughing. Providers in the high-information group were significantly less likely to ask how the patient feels in general. One might have expected that providers are more likely to conduct respiratory examinations with SPs in the control group – to try and identify physical symptoms of TB given the less clear-cut introductory statement. However, we find no significant differences in the types of physical examinations performed.

**Fig. 3:**
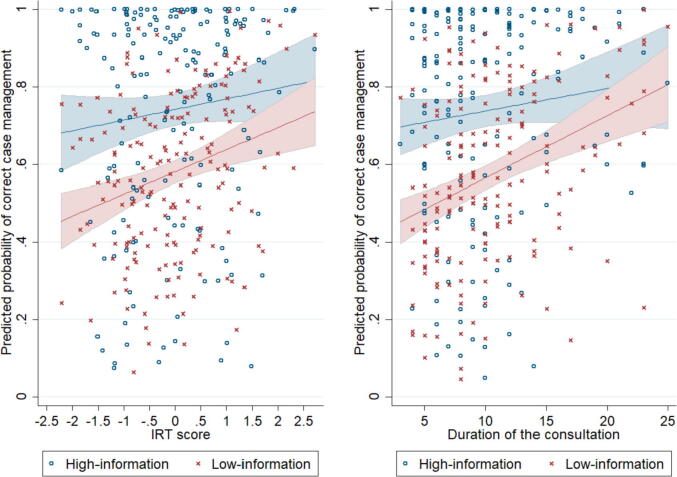
Correlation between correct case management and provider effort, by treatment.

Notes: We regressed the quality of case management on provider effort, separately for patients in the treatment and control group. In the graph on the left, effort is measured by the IRT score (see Appendix A3 for details). On the right hand side, we use duration of the consultation (in minutes). We use probit regressions that control for facility characteristics (see Table 1) and SP fixed effects. The blue dots show consultation-level predicted values for the high-information group. The red crosses show consultation-level predicted values for the low-information group. The corresponding blue and red lines show the fitted values with 95% confidence intervals.

To illustrate the relative roles of provider and patient inputs in determining the quality of care in medical consultations, we regress the probability of correct case management on two measures of provider effort separately for patients who disclose more or less information. The predicted probabilities are presented in Fig. 3. The graphs show that when patients start the consultation by disclosing more symptoms (blue dots), the level of output (quality of care) reached at the end of the consultation is systematically higher, unless providers exert high levels of effort. In other words, with more initial input from patients, the output of the consultation production function is higher.

### 5.3 Exploring mechanisms behind providers’ low effort

Why is it that providers do not ask their patients relevant questions? This section examines three plausible explanations: lack of knowledge, resource constraints in the health system and low motivation.

To test whether lack of knowledge explains the inability of providers to adjust their level of effort during the consultation and ask more relevant questions, we use data from a clinical vignette presenting the same TB case to a subset of 119 providers. Overall, 81% of providers (*n* = 96) correctly managed the hypothetical patient in the vignette. Strikingly, amongst the providers who did *not* manage the SP correctly in practice, the vast majority (89%) were able to correctly diagnose and manage the hypothetical patient. This finding suggests that substantial “know-do gaps”, rather than lack of knowledge are behind the poor performance in practice.

Mirroring the SP experiment, in the vignette survey providers were asked about either a hypothetical patient using the short introduction to describe their symptoms of TB (low-information vignette, *n* = 67) or the longer introduction (high-information vignette, *n* = 52) – see Appendix A7 for more details on the vignette experiment, including evidence that the two groups are balanced on observable characteristics (Appendix Table A7). Unlike what we found in the SP experiment, the probability of correct case management in the vignette experiment is not influenced by the amount of information received by providers at the start of the vignette (Appendix Table A8). Unlike what happens in practice, most providers who complete the low-information vignette seek more information about the patient’s symptoms and are thereby able to “catch up”; 66% of providers ask hypothetical patients about either weight loss or blood in sputum (the symptoms volunteered in the treatment group). This result confirms that lack of clinical knowledge does not seem to explain our results, since providers *know* how to ask relevant information related to the presenting complaint and make up for differences in information provided by patients. When testing for heterogeneity of the treatment effect by provider knowledge, we find no evidence that providers with higher knowledge provide better quality of care (Appendix Table A9). This result further supports the notion that provider knowledge does not explain low provider performance.

Another possible explanation for our findings is that providers’ behaviours reflect a response to the constraints they face – either personally (time constraints) or more broadly they internalise the resource constraints of the health system, and learn to give priority to cases appearing more serious or urgent.^3^ Hence, in our experiment, providers may prefer to be more decisive with clear cases of TB (i.e. in the high-information group) and send them immediately for a referral or TB test, whilst not investigating much further cases appearing less serious and advising patients to monitor their symptoms. We can test this hypothesis by looking at whether SPs in the control group are more likely to be asked to “come back if symptoms worsen” or “come back if there is blood in their sputum”. Providers ask SPs to return in case of worsening symptoms in only *n* = 29 consultations (15%) across treatments, and we find no evidence that control patients, disclosing less serious symptoms, are more likely to be told to return if symptoms worsen (Appendix Table A4). This result does not seem to support the notion that providers in the low-information group are responding to resource constraints – and indeed, the volume of patients visiting these facilities is low (see section 4.3), suggesting that providers do not face sharp time constraints and that the system could cope with the demand.

Finally, we examine whether provider motivation, proxied by providers’ self-reported intention to quit the facility (available for *n* = 134 observations), can explain low effort. We find some evidence that the benefit of symptom disclosure on the quality of care is moderated by provider motivation, as the effect becomes no longer significant once the interaction term is included in the specification – see Table A10 in Appendix. This result suggests that the treatment effect is concentrated in providers with low levels of motivation. Indeed, for providers with low motivation, the probability of correct management is 40 percentage points higher when they receive more information initially (a 83% increase), compared to only 9 percentage points (13% increase) for providers with high motivation^4^. However, it is unclear what behavioural mechanism lies behind this effect. As shown in Appendix Table A11, we do not find evidence that providers with low motivation exert less effort than other providers.

## 6 Robustness checks

A potential concern with the design is that patients who report more symptoms could be perceived as more empowered or educated – making providers work harder, perhaps because they feel that more vocal patients are more likely to hold them accountable. Accountability is generally very limited in healthcare markets in LMICs and previous work has shown that introducing even soft accountability mechanisms such as community involvement can improve healthcare provision (Björkman and Svensson, 2009, Fox, 2015, Hernández et al., 2019).

Our sense is that, in our setting, the potential effect of patients being perceived as more empowered is unlikely to be substantial for several reasons. To begin, although SPs in the treatment group offered a little more information at the start of the consultation, they were trained to answer other questions in the same manner. Perhaps more importantly, because the treatment was randomised at the SP level (meaning that each fieldworker played both roles) other indicators of empowerment or status – such as demeanour, clothes, or accent – are held constant. We can test whether providers behaved differently towards patients in the high-information group – focusing on providers’ bedside manner and behaviours that are easily observable by patients. SPs indicated the extent to which they felt that providers were attentive, cared about their problems, listened to what they were saying, looked at them whilst they spoke, were unkind to them, explained things well or made them feel uncomfortable. They also recorded whether providers were distracted during the consultation, specifically whether they spoke on the phone or whether they spoke to other patients. It is likely that such provider behaviours would be sensitive to the perception that patients are particularly empowered or educated, as providers would be more courteous. As shown in Appendix Table A12, we find no significant differences along any of these dimensions of provider behaviour, offering no support for the notion that patients who volunteered more information were perceived as more empowered.

Second, one of the key assumptions of the SP methodology is that providers treat SPs as if they were real patients. We verify whether our main results hold when the 17 consultations in which providers suspected that SPs were not real patients are excluded from the analysis. As shown in Appendix Table A13 i, results are robust to the exclusion of these patients and overall, the treatment effect is larger in magnitude among this sample.

Third, to alleviate concerns that null results are driven by low power, we increase the precision of our estimates by controlling for a range of measures of provider knowledge (whether providers correctly managed the TB vignette, a competence index capturing the weighted proportion of questions asked and physical examinations completed in the TB vignette, the proportion of all vignettes managed correctly, correct management in the dysentery, malaria, pneumonia, asthma and angina vignette). Tables A14 to A16 include the full set of controls, whilst estimations reported in Tables A17 to A21 use the lasso estimator to select the controls following a PDS methodology (Belloni et al., 2013). Our results remain largely unchanged when providers’ ability to correctly manage the case is accounted for, although effect sizes are somewhat larger than in our main analysis.

Finally, we reproduce the main analysis using maximum likelihood estimation or Firth logistic regressions (Table A22) as well as probit regressions (Table A23). We find that results are robust to such specifications and are largely of the same magnitude.

## 7 Discussion and conclusion

Our study reports the results of a simple experiment where standardised patients were randomised to present more or less information about their TB symptoms at the start of the consultation. The results suggest that patients in Senegal can improve the quality of medical care by volunteering more information about their symptoms. This is despite reasonably good levels of quality of care for TB in the study setting, which is considerably higher than what has been found in India (Das et al., 2015) and in Kenya (Daniels et al., 2017)^5^, as 60% of ‘typical’ patients, volunteering relatively little information, are correctly managed. Nonetheless, quality of care is significantly better for patients who disclose two more symptoms of TB, as 76% of them end up correctly managed – an improvement of 27%.

Our results also underscore that provider effort remains a significant barrier to higher quality healthcare. We find that providers do not adequately take patients’ histories and fail to ask for the key pieces of information needed to make a correct diagnosis. This is in line with recent studies suggesting that poor quality healthcare in LMICs is partially due to providers not investing enough effort (Gertler and Vermeersch, 2013, Leonard and Masatu, 2010b, Leonard and Masatu, 2010a, Mohanan et al., 2015, Sylvia et al., 2017). We add to this body of work by showing that provider effort is largely independent of patient effort (i.e. symptom disclosure) – even though provider and patient inputs are complementary to reach high levels of quality of care. In line with previous work (Leonard and Masatu, 2010b, Leonard and Masatu, 2010a, Mohanan et al., 2015), we find evidence of the existence of know-do gaps, as providers’ failure to invest effort in consultations is not driven by a lack of knowledge. With a simple vignette experiment, we even show that providers have the necessary clinical skills required to take the history of a hypothetical patient volunteering little information but fail to do this in practice. We also rule out the idea that providers may treat the two types of patients differently because they internalise constraints on the supply-side (limited resources in the health system) or on the demand-side (patients’ financial constraints). Instead, we find some evidence that low effort in practice is due to low motivation. Although we are unable to clearly identify the mechanism at play, it is possible that, instead of adapting their effort to the patient case, demotivated providers apply a simple heuristic based on the number of symptoms volunteered. For instance, patients volunteering few symptoms are classified as suffering from a mild condition (and providers deem unnecessary to rule out serious illnesses), while those divulging several symptoms are categorised as more serious cases.

Our results have two main policy implications. First, our findings suggest that interventions encouraging more symptom disclosure by patients during the consultation, arguably a costless act for patients, could represent new ways to improve the quality of care in settings where alternative strategies have failed. It would be important to test the scalability of our findings, by evaluating information campaigns that empower patients to volunteer information about their symptoms to health providers. Although the onus for receiving correct treatments should not be on the patient, such strategies could help improve quality of care, despite existing supply-side constraints which have proven to be hard to address. Public awareness campaigns about specific diseases are not uncommon in LMICs (see for example, Price, 2013, Sharma and Sharma, 2007). In the case of TB, these often focus on modes of transmission, danger signs and the cost of treatment – primarily to encourage care seeking (Yadav and Rawal, 2016). Our results suggest that these campaigns should potentially also encourage patients to *tell* providers about specific symptoms. Such an approach could also be suitable for other life-threatening conditions that have very specific symptoms, such as meningitis, asthma or cardiac problems.^6^ Whilst in our view such an approach is promising, several issues would need to be considered. One is that patients in low-income settings often describe providers as authoritarian or frightening figures dominating the consultation process (Kwame and Petrucka, 2020, Mannava et al., 2015), so that it is unclear whether patients would be comfortable taking up a more active role. Furthermore, information campaigns could exacerbate inequalities in access to quality care if more educated or empowered patients were better able to present comprehensively their chief complaint.

Second, our results confirm that limited provider effort remains a key constraint to attain higher quality of care. We find that even though providers have sufficient knowledge and enough time, they fail to ask the right questions. Although changing the incentives faced by public providers could *a priori* increase effort, so far there is limited evidence that performance-based incentives can increase quality of care (Das et al., 2016, Das et al., 2016, Diaconu et al., 2021) – potentially due to information asymmetry in healthcare markets, which limits the potential benefits of competitive incentives (Das et al., 2016, Das et al., 2016, Das and Hammer, 2014). An alternative strategy to ensure that providers ask patients the right questions could be to improve their history-taking skills. Although providers might have sufficient clinical knowledge, most front-line primary care providers (i.e. nursing assistants, nurses and midwives) have not received specific medical training on how to interview patients and take their history to arrive at a diagnosis in a systematic way. Even though providers managed to show good history-taking skills in hypothetical vignettes, the know-do gap in that area could come from the limited emphasis given to those skills in their initial training, and how this may have shaped their perception of their role as providers and their interactions with patients during consultations. Hence, training workshops on the importance and art of history-taking – such as the ones being widely implemented in high-income settings (Keifenheim et al., 2015) – could improve the quality of care. Another option would be to equip providers with decision support systems, prompting them to ask specific questions or conduct specific examinations (Agarwal et al., 2018). For example, a recent study in Burkina Faso found that providers using tablet-based decision aids completed 30% more clinically relevant actions in consultations with sick children than those using paper-based or no decision aids, although the intervention did not improve the quality of case management (Cousens et al., 2018).

There are some limitations to our study. A first limitation is that, because enumerators acting as SPs are in fact in good health, they might not “look like” most TB patients. This creates a potential for bias as, based solely on initial facial observation, providers might judge that SPs are not very ill. Because of an anchoring effect (Friedlander & Stockman, 1983), providers might perceptually lock on this initial judgement and fail to adjust it when more information is offered. We tried to counteract this by selecting SPs who did not look particularly healthy – SPs were slightly underweight or very slim and did not have a “healthy glow”. In addition, within the study setting demand for healthcare is low. What emerged from discussions with providers in study areas is that providers consider that patients often delay care seeking and consult them when they are already seriously ill – a perception which is in line with the broader literature (Getnet et al., 2017). If providers assume that patients who come to see them are truly unwell, this should counter-balance the anchoring effect of the relatively healthy-looking SPs.

A second limitation relates to the external validity of our results. First, the effect size likely represents an upper-bound estimate of the effect of what could be achieved by an intervention targeting patients’ communication to improve quality of care. This is because SPs volunteered relevant symptoms in a clear manner, following a specifically designed and rehearsed scenario – which is unlikely to be representative of real patients’ behaviour. Second, the study only uses one clinical case, which presents with very clear symptoms. It is unclear if the same results could be obtained for ailments presenting with less obvious signs (e.g. malaria or pneumonia). Nonetheless, several other life-threatening illnesses usually manifest themselves through specific signs which, if shared with providers, could probably reduce the likelihood of misdiagnosis. More generally, the ambition of the study is not to claim that the results are externally valid. Instead, it is to present a proof-of-concept evaluation for a potential new avenue to improve quality of care – one that has been neglected in academic and policy circles alike. To that end, the fact that the effect size observed is very large provides a strong basis for testing this idea in a less controlled and more realistic setting.

This study makes an important contribution to the academic literature and current policy debates on the low performance of providers in LMICs. It provides valuable proof-of-concept evidence that one way to improve quality of care is by ensuring that patients can more effectively relay information about their symptoms to providers. In doing so, we open potential avenues for future research into the possible ways in which this can be best achieved. Studies are needed to test the effects of awareness or information campaigns targeting patients and encouraging them to share more relevant information. At the same time, it would be interesting to compare these studies to more standard interventions targeting providers and improving their communication and history-taking skills.

### CRediT authorship contribution statement

**Roxanne J. Kovacs:** Conceptualization, Validation, Formal analysis, Investigation, Data curation, Writing – original draft, Writing – review & editing. **Mylene Lagarde:** Conceptualization, Investigation, Funding acquisition, Writing – original draft, Writing – review & editing, Supervision. **John Cairns:** Writing – review & editing.

## Declaration of Competing Interest

The authors declare that they have no known competing financial interests or personal relationships that could have appeared to influence the work reported in this paper.

## Footnotes

1 Providers in the control group would be expected to ask about blood in the sputum and weight loss, but not those in the treatment group who received this information. Hence these two items enter in the construction of measures for providers in the control group, but not for those in the high-information arm.

2 All results are generally robust to using a PDS lasso approach to select controls – see robustness checks.

3 Another hypothesis is that providers internalise their *patients’* constraints (financial or other) to travel to another facility to do a screening test. This would lead to the same behaviour, of only referring those they see as more urgent, and telling others to monitor their symptoms.

4 The predicted probability of correct case management, among high-motivation providers, is 81% in the high-information group and 72% in the low-information group. In comparison, the predicted probability of correct case management, among low-motivation providers, is 88% in the high-information group and 48% in the low-information group.

5 12% of Indian providers manage the case correctly and 50% of providers in Kenya.

6 Several serious conditions often present with very specific symptoms. For example, patients suffering from meningitis will generally have a stiff neck and photophobia, and sometimes a non-blanching rash, especially children. Asthma is often characterised by a high-pitched whistling noise (wheezing) when patients breathe out. Individuals suffering from cardiac failures will have a very sharp pain, which often irradiates in the left arm. Early in the pandemic, information campaigns highlighted lack of taste and smell as a characteristic sign of Covid-19, in addition to the less specific symptoms of cough and fever.
