# Supplementary material for: Can patients improve the quality of care they receive? Experimental evidence from Senegal
Source: World Dev. 2022 Feb;150:105740. doi: 10.1016/j.worlddev.2021.105740 (PMC8651629; doi:10.1016/j.worlddev.2021.105740)
Supplement: Supplementary data 1 [file mmc1.pdf]

Online Appendix

This document includes supplementary material for the manuscript "Can patients improve the quality of care they receive? Experimental evidence from Senegal".

A.1 Study areas 2

Figure A1 – Map of study areas 2

A.2 The standardised patient case 3

Debriefing checklist 3

SP case overview 5

A.3 Measuring provider effort 8

Data 8

Item Response Theory 9

Table A1 – IRT analysis for the low-information group 10

Table A2 – IRT analysis for the high-information group 11

Figure A2 – Correlation between correct case management and provider effort, by treatment 13

A.4 Measuring structural quality 14

A.5 Additional analyses 16

Table A3 – Treatment effects on TB specific case management (sputum test) 16

Table A4 – Treatment effects on symptom monitoring advice to patients 16

A.6 Differences in effort in the consultation across treatments 17

Table A5 – Questions asked by providers 17

Table A6 – Physical examinations done by providers 17

A.7 Vignette experiment 18

Table A7– Balance checks for vignette experiment 18

Table A8 – Effect of information on performance in vignettes 19

A.8 Heterogeneous effects 20

Table A9 – Heterogeneous treatment effects by provider knowledge 20

Table A10 – Heterogeneous treatment effects on correct management by provider motivation 21

Table A11 – Heterogeneous treatment effects of effort by provider motivation 22

A.9 Robustness checks 23

A.9.1 Alternative mechanisms 23

Table A12 – Differences in providers' bedside manners across treatment groups 23

Table A13 – Treatment effects on correct case management (undetected SPs only) 23

A.9.2 Increasing precision of estimates 24

Table A14 – Treatment effects on quality of care (knowledge controls) 24

Table A15 – Treatment effects on provider effort 25

Table A16 – Treatment effects on the duration of the consultation 26

Table A17– Treatment effects on correct case management (PDS lasso) 27

Table A18 – Treatment effects on the duration of the consultation (PDS lasso) 28

Table A19 – Treatment effect on provider effort (PDS lasso) 29

Table A20 – Heterogeneous treatment effects by provider motivation (PDS lasso) 30

Table A21 – Treatment effects on provider effort (PDS lasso) 30

A.9.3 Alternative specifications 32

Table A22 – Effect of patient attitude on correct case management (firth logistic regressions) 32

Table A23 – Effect of patient attitude on correct case management (probit regressions) 32

A.1 Study areas

Data were collected in a representative sample of facilities in four rural regions of the country (Ziguinchor, Sédhiou, Tambacounda, Kédougou). The map below shows the administrative boundaries for all regions of the country. For study regions, boundaries for health districts are shown.

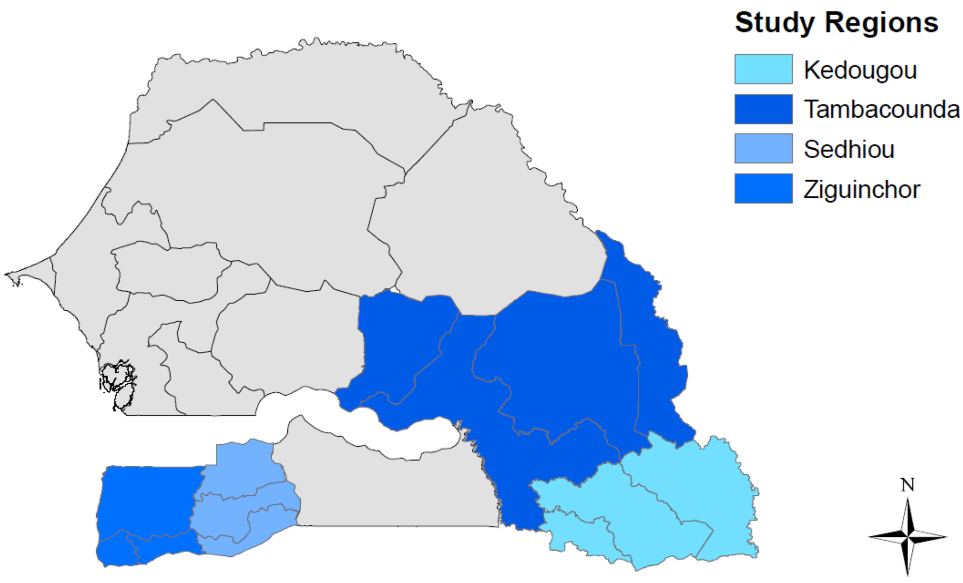

Figure A1 – Map of study areas

A.2 The standardised patient case

Debriefing checklist

Within one hour of their consultation, SPs had to complete a debriefing In a first section, SPs were asked about:

- Arrival time at the facility
- Number of patients waiting when they arrived
- Start time of the consultation
- Duration of consultation (in minutes)
- Number of patients waiting when they left the facility

In the next section, SPs were asked to indicate which questions had been asked by providers during the consultation, amongst the following:

1. Do you have trouble breathing?
2. For how long exactly have you been coughing?
3. Is the cough productive/ is there sputum?
4. Is there blood in the sputum?
5. Do you have chest pain?
6. Do you cough all day long?
7. Does anybody in your surroundings have the same cough?
8. Did you have fever?
9. Do you have night sweats?
10. Do you have chills?
11. Did you lose weight?
12. Do you eat normally/ Do you have an appetite?
13. How do you feel in general?
14. Do other people in your family cough a lot or have asthma?
15. What is your job?
16. Do you have any other pains or symptoms?
17. Did you take any medication?
18. Are you exposed to cigarette smoke or do you use incense?

Then the questionnaire asked SPs to indicate which physical examinations had been performed amongst the following:

1. Take temperature
2. Take blood pressure
3. Take pulse
4. Take respiratory frequency
5. Use stethoscope at the front
6. Use stethoscope at the back

7. Do rapid diagnostic test for malaria

In this section SPs indicated the recommendations made by providers.

1. Use a tissue and don't spit on the ground
2. Come back if there is more blood in sputum
3. Take prescribed medication
4. Come back with laboratory results
5. Come back if symptoms worsen
6. Drink a lot to facilitate expectoration

SPs were asked to indicate how much they paid for the consultation. Finally, the debriefing questionnaire included a section on drugs dispensed or prescribed. This section was completed by team leaders, who were trained health professionals (nurses and midwives) – based on information retrieved from prescriptions given to SPs or drugs dispensed by providers. All drugs provided, and prescriptions given to SPs were photographed. Team leaders listed all of the drugs that were given or prescribed. The same was done for any tests or follow-up exams.

SP case overview

SPs were given a detailed script that documents their personal and medical history, which is shown below.

- **Personal history**

You are called [your real name]. You are x years old [your real age]. You have X sisters and X brothers [like in real life]. You are married and have X children.

Your job:

You work on buildings sites in Dakar as a mechanic. When you do that, you live in a room with other builders (8 per room).

You have just come back after working in Dakar for 2 months, repairing cars. You were filling in for somebody and now you are waiting for the next building site to start.

How you arrived in the local area:

In the village, you live with a cousin that you wanted to visit because the building site you were working in in Dakar is completed.

- **Medical history**

Your health in general:

You don't drink, you don't smoke, and you did not have any specific health problems in the past (except for malaria, which you contracted several time). Everyone in your family is globally in good health, both your parents are alive and in good health and your brothers and sisters are also in good health.

The problem that brings you to the facility:

In the past 2 weeks, you have not done much because you felt very tired, you did not really have an appetite and you coughed a lot. For about a week now, you wake up at night, sweating. You thought that this would pass on its own, which is why you did not go to see a doctor before, but your wife told you to come because you were not getting better.

- **Standardised answers to questions**

SPs were trained to give standardised answers to a list of questions that were providers were likely to ask. During the training workshop, SPs were taught to provide exactly these responses. SPs were also taught to improvise in cases where providers asked unexpected questions. Whenever SPs were asked about a symptom or problem not listed below, they indicated that they did not have the symptom or did not experience the problem.

QUESTION	ANSWER
Do you have trouble breathing?	No
For how long have you been coughing?	2 weeks
Is the cough dry or productive?	It is productive
Is there sputum?	Yes
Is there blood in the sputum?	Yes
Describe the sputum to me	It is a bit yellow
Do you have chest pain?	Sometimes
Do you cough all day long?	Yes, I cough a lot
Is the cough worse in the morning?	Yes, when I wake up it is worst
Does somebody else in your house have the same cough?	No, I don't think so
Do you use incense or are you exposed to cigarette smoke?	I don't smoke, and I don't use incense
Did you have fever?	Yes, a bit I think, at night
Do you have night sweats?	Yes, my sheets are sometimes humid when I wake up
Do you have chills?	Yes, a bit sometimes
Did you lose weight?	Yes, the trousers that were once tight on me don't really fit anymore
How much do you weigh?	I don't know exactly
How much weight did you lose?	I don't know really, but my clothes feel bigger
Did you lose your appetite?	Yes, I don't eat a lot and I am not really hungry
Do you drink alcohol?	No

Do you smoke?	No
How do you feel in general?	Very tired and weak
Do other people in your family cough a lot or have asthma?	No
What do you do for a living?	I am a mechanic, I work on building sites around Dakar
Do you have any other symptoms/ health problems?	No, I just feel very tired and exhausted
Did you take any medication?	No
Why did you not come to the facility before?	I thought that this would pass on its own
Why are you coming now?	I feel really tired and I cannot work. My wife and my friends told me to come.
Did you consult someone else (traditional healer, another provider) before?	No
How do you live?	In Dakar, I share a room with 8 people. In the village, I live with my family.
Is the place you live well aerated?	Not in Dakar, but in the village.

A.3 Measuring provider effort

Data

We use three measures of provider effort: an index score based on IRT, the proportion of relevant history questions asked and physical examinations performed and the duration of the consultation.

The IRT score as well as the simple score capturing the proportion of items done are based on the history questions and physical examinations shown below. As providers in the high-information (treatment group) are told by patients that they have blood in sputum and have lost weight, these two items are not used for calculating effort for these providers.

- History questions:
  - Blood in sputum?
  - People in surrounding cough?
  - Weight loss?
  - Cough or asthma in family?
  - Chest pain?
  - Trouble breathing?
  - Night sweats?
  - Loss of appetite?
  - Exposed to cigarette smoke?
  - Chills?
  - Duration of cough?
  - Productive cough?
  - Cough lasts all day?
  - Fever?
  - Any other symptoms
  - How do you feel in general?
- Physical examinations:
  - Take temperature
  - Take blood pressure
  - Take pulse
  - Take respiratory frequency
  - Use stethoscope on front
  - Use stethoscope on back

1. Methods

Item response theory (IRT), first applied in the context of psychometrics and educational testing, is a model-based approach to developing and scoring tests (or checklists) for estimating latent individual traits (such as ability, or in this case, effort). Using maximum likelihood methods, the IRT score assigns greater weight to items that are more difficult (less likely to be completed) and that discriminate better among providers in terms of their unobserved effort (Das and Hammer 2005).

In this application, employ a two-parameter ('2pl') logistic function for modelling the probabilities that providers successfully complete individual checklist items, given their unobserved effort level, using the *irt 2pl* command in STATA (STATA, 2021). The two-parameter approach allows for variation in difficulty and discrimination levels. In our analysis, items (history questions and physical examinations) are binary, in the sense that they are either correct (1 = completed by the provider) or incorrect (0 = not completed by the provider). Items are assumed to be equally discriminating and only vary in terms of their level of difficulty.

In constructing the IRT score, we separately run the analysis for providers in the treatment and the control group. We exclude the items that were manipulated in the experiment for providers in the treatment group, to avoid unnecessarily rewarding (or punishing) providers for (not) asking for information they already received from patients. All other items listed in Appendix A2 are included.

As described in further detail in STATA (2021), we assume that Y_{ij} is the yet to be observed outcome for item i from person j – where y_{ij} is the observed value of Y_{ij}. When $y_{ij} = 1$ this is referred to as correct, whilst $y_{ij} = 0$ as incorrect.

Based on the IRT parameterisation, the probability of person j with latent trait level θ_j (clinical competence) providing a correct response to item i, is given by (STATA, 2021, p.37):

$$\Pr(Y_{ij} = 1|a, b_i, \theta_j) = \frac{\exp\{a(\theta_j - b_i)\}}{1 + \exp\{a(\theta_j - b_i)\}}$$

Here, a refers to discrimination and b to the difficulty of the item. To identify these parameters, we fit a model using the slope-intercept form (STATA, 2021, p.37).

2. Results

The table below shows output from IRT analysis for the low-information group.

Table A1 – IRT analysis for the low-information group

	Coef.	Std. Err.	z	P>z	95% CI	
**Discrimination**						
Cough or asthma in family?	16.92	22.33	0.76	0.45	-26.85	60.68
People in surrounding cough?	3.95	2.89	1.37	0.17	-1.71	9.61
Weight loss?	1.27	0.57	2.23	0.03	0.15	2.39
Respiratory frequency	1.17	0.64	1.82	0.07	-0.09	2.43
Use stethoscope on front	1.03	0.45	2.30	0.02	0.15	1.91
Exposed to cigarette smoke?	0.96	0.47	2.05	0.04	0.04	1.88
Loss of appetite?	0.92	0.46	1.99	0.05	0.01	1.82
Use stethoscope on back	0.84	0.41	2.06	0.04	0.04	1.64
Take blood pressure	0.82	0.44	1.86	0.06	-0.05	1.69
Blood in sputum?	0.77	0.39	1.97	0.05	0.00	1.54
Take temperature	0.60	0.42	1.41	0.16	-0.23	1.42
Cough all day?	0.58	0.34	1.70	0.09	-0.09	1.26
Night sweats?	0.58	0.40	1.45	0.15	-0.20	1.37
Duration of cough?	0.41	0.35	1.17	0.24	-0.28	1.11
Take pulse	0.35	0.34	1.03	0.30	-0.31	1.00
Productive cough?	0.31	0.30	1.02	0.31	-0.28	0.89
Fever?	0.27	0.30	0.90	0.37	-0.32	0.87
Chest pain?	0.05	0.42	0.11	0.91	-0.77	0.86
Chills?	0.01	0.51	0.02	0.99	-0.98	1.00
How do you feel in general?	-0.02	0.30	-0.07	0.95	-0.60	0.56
Any other symptoms?	-0.08	0.28	-0.27	0.79	-0.62	0.47
Trouble breathing?	-0.55	0.75	-0.73	0.47	-2.02	0.92
	Coef.	Std. Err.	z	P>z	95% CI	
**Difficulty**						
How do you feel in general?	-37.26	547.20	-0.07	0.95	-1109.75	1035.23
Trouble breathing?	-6.00	7.57	-0.79	0.43	-20.84	8.84
Duration of cough?	-3.40	2.77	-1.23	0.22	-8.82	2.03
Take temperature	-2.93	1.88	-1.56	0.12	-6.61	0.76
Fever?	-2.31	2.58	-0.90	0.37	-7.36	2.75
Take blood pressure	-1.87	0.86	-2.18	0.03	-3.55	-0.19
Any other symptoms?	-1.38	5.68	-0.24	0.81	-12.51	9.76
Productive cough?	-0.36	0.75	-0.48	0.63	-1.82	1.11
Use stethoscope on front	0.88	0.38	2.32	0.02	0.14	1.62
Weight loss?	1.18	0.42	2.80	0.01	0.35	2.00
Use stethoscope on back	1.22	0.55	2.20	0.03	0.13	2.30
People in surrounding cough?	1.34	0.26	5.18	0.00	0.83	1.84
Blood in sputum?	1.45	0.69	2.11	0.04	0.10	2.80
Cough or asthma in family?	1.60	0.26	6.08	0.00	1.09	2.12
Loss of appetite?	1.62	0.71	2.29	0.02	0.24	3.00
Cough all day?	1.93	1.09	1.77	0.08	-0.21	4.07
Exposed to cigarette smoke?	2.05	0.82	2.48	0.01	0.43	3.66
Respiratory frequency	2.60	1.09	2.38	0.02	0.46	4.74
Night sweats?	2.70	1.73	1.56	0.12	-0.69	6.10
Take pulse	3.44	3.26	1.06	0.29	-2.94	9.82
Chest pain?	42.72	387.91	0.11	0.91	-717.57	803.01
Chills?	263.10	15339.75	0.02	0.99	-29802.25	30328.45

Table A2 – IRT analysis for the high-information group

	Coef.	Std. Err.	z	P>z	95% CI	
**Discrimination**						
Use stethoscope on front	3.89	2.13	1.82	0.07	-0.29	8.07
Use stethoscope on back	2.60	1.01	2.58	0.01	0.62	4.57
Night sweats?	1.20	0.49	2.44	0.02	0.24	2.17
How do you feel in general?	1.19	0.53	2.25	0.03	0.15	2.24
Loss of appetite?	1.00	0.42	2.40	0.02	0.18	1.82
Chest pain?	0.95	0.42	2.30	0.02	0.14	1.77
Take blood pressure	0.94	0.37	2.51	0.01	0.21	1.67
Fever?	0.89	0.32	2.76	0.01	0.26	1.52
Productive cough?	0.86	0.32	2.72	0.01	0.24	1.49
Take temperature	0.82	0.38	2.14	0.03	0.07	1.58
People in surrounding cough?	0.81	0.38	2.13	0.03	0.06	1.55
Cough or asthma in family?	0.77	0.48	1.60	0.11	-0.17	1.70
Respiratory frequency	0.67	0.57	1.19	0.23	-0.44	1.78
Duration of cough?	0.60	0.31	1.95	0.05	0.00	1.20
Exposed to cigarette smoke?	0.48	0.40	1.20	0.23	-0.31	1.27
Cough all day?	0.39	0.36	1.08	0.28	-0.31	1.08
Trouble breathing?	0.35	0.57	0.62	0.54	-0.76	1.47
Chills?	0.15	0.57	0.26	0.79	-0.97	1.27
Any other symptoms?	0.12	0.25	0.50	0.62	-0.37	0.62
Take pulse	0.02	0.28	0.08	0.94	-0.53	0.58
	Coef.	Std. Err.	z	P>z	95% CI	
**Difficulty**						
Take temperature	-2.28	0.92	-2.48	0.01	-4.08	-0.47
Duration of cough?	-1.79	0.90	-2.00	0.05	-3.55	-0.04
Take blood pressure	-1.51	0.54	-2.81	0.01	-2.56	-0.46
Fever?	-0.20	0.27	-0.73	0.47	-0.73	0.34
Productive cough?	0.39	0.29	1.36	0.17	-0.17	0.96
Use stethoscope on front	0.58	0.15	3.95	0.00	0.29	0.87
Use stethoscope on back	0.76	0.18	4.22	0.00	0.41	1.12
Night sweats?	1.59	0.50	3.19	0.00	0.61	2.56
People in surrounding cough?	1.62	0.68	2.39	0.02	0.29	2.95
Loss of appetite?	1.72	0.60	2.90	0.00	0.56	2.89
Chest pain?	1.94	0.70	2.76	0.01	0.56	3.32
How do you feel in general?	1.97	0.66	3.00	0.00	0.68	3.25
Cough or asthma in family?	3.01	1.62	1.85	0.06	-0.18	6.19
Exposed to cigarette smoke?	4.31	3.38	1.28	0.20	-2.31	10.93
Respiratory frequency	4.37	3.30	1.32	0.19	-2.10	10.83
Cough all day?	4.43	3.93	1.13	0.26	-3.27	12.13
Any other symptoms?	4.62	9.34	0.49	0.62	-13.68	22.93
Trouble breathing?	8.55	13.45	0.64	0.53	-17.81	34.90
Chills?	19.67	74.54	0.26	0.79	-126.43	165.77
Take pulse	49.98	644.11	0.08	0.94	-1212.45	1312.41

3. Restricting analysis to most discriminating items

We construct an alternative version of Figure 3 in the main text, where we re-measure effort as the proportion of items done by providers, including only the most discriminatory 50% of items identified through IRT analysis presented above. We regress the quality of case management on provider effort, separately for patients in the treatment and control group and plot predicted probabilities.

The left-hand graph in Figure A2 measures effort as the proportion of items done by providers, including only the most discriminatory half of items, identified through IRT analysis. The right-hand graph in Figure A2 uses the duration of the consultation.

The list of items identified as most-discriminatory by the IRT analysis for the low-information group are:

- Loss of appetite?
- Take temperature
- Take blood pressure
- Use stethoscope on front
- Use stethoscope on back
- Blood in sputum?
- People in surrounding cough?
- Weight loss?
- Cough or asthma in family?
- Exposed to cigarette smoke?
- Take respiratory frequency

The list of items identified as most-discriminatory by the IRT analysis for the high-information group are:

- Loss of appetite?
- Take temperature
- Take blood pressure
- Use stethoscope on front
- Use stethoscope on back
- Chest pain?
- Night sweats?
- Productive cough?
- Fever?
- How do you feel in general?

Figure A2 – Correlation between correct case management and provider effort, by treatment

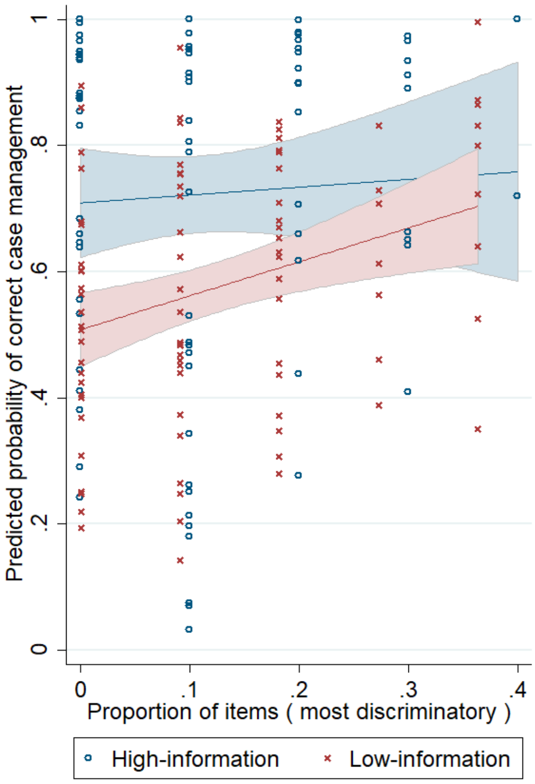

Reference

STATA (2021) *irt 2pl-Two-parameter logistic model.*

A.4 Measuring structural quality

We create a simple aggregate score of structural quality that indicates the proportion of essential drugs and equipment available in facilities. The list of essential drugs and equipment is shown below.

- **Equipment:**

1. Forceps
2. Glucometer
3. Beds for patients / consultations
4. Microscope
5. Fridge
6. Speculum
7. Obstetrical stethoscope
8. Stethoscope
9. Table to work on (for provider)
10. Sphygmomanometers
11. Thermometer
12. Suction cup
13. Stem (infusion port)
14. Box of surgical instruments
15. Antiseptic solution (decontamination solution)
16. Gloves
17. Sterile gloves
18. Cotton or sterile compresses
19. Childbirth kit
20. Urinary test strips
21. Rapid diagnostic test for malaria
22. Rapid diagnostic test for HIV
23. Autoclave
24. Measuring tape
25. Syringe
26. Catheter
27. Syringe driver
28. Pregnancy test

- **Drugs:**

1. Glucose serum
2. Saline solution
3. Haemocoel solution
4. Ringer lactate
5. Paracetamol syrup / tablets
6. Aspirin tablets
7. Chloroquine
8. Ceftriaxone
9. ACT (Combined Artemisinin Therapy)
10. Antihypertensive
11. Quinine perfusion
12. Artemether injection
13. Artesunate injection
14. Cefixime
15. Apo-Amoxi Clav
16. Corticosteroid
17. Amoxycillin
18. Cotrimoxazole
19. Metronidazole
20. Penicillin Extencillin / Benzathine
21. Doxycycline
22. Mbendazole / Albendazole
23. Expectorant (Carbocisteine, ...)
24. Magnesium sulfate
25. Oxytocin
26. Ergometrine / methergine / misoprostol
27. Vitamin K
28. Vitamin A
29. Antihistamines
30. Iron/ Folic Acid
31. Ciprofloxacin
32. Diazepam

33. Sulfoxide pyrimethamine (SP) / Intermittent preventive treatment of malaria
34. Salbutamol
35. Insecticide-treated mosquito nets
36. Hepatitis Vaccine
37. BCG vaccine (tuberculosis)
38. Pentavalent vaccine (diphtheria, tetanus, pertussis, haemophilus influenza and hepatitis B)
39. RR Vaccine (measles - rubella)
40. Vaccine VPI (polio)
41. PCV13 (conjugate pneumococcal vaccine)
42. Rotavirus vaccine
43. Ibuprofen
44. Zinc
45. Vogalene

A.5 Additional analyses

Table A3 – Treatment effects on TB specific case management (sputum test)

	(1)	(2)	(3)	(4)
Patient discloses more information	0.144**	0.140**	0.143**	0.134**
	(0.069)	(0.069)	(0.069)	(0.065)
Facility characteristics	No	Yes	Yes	Yes†
Provider characteristics	No	No	Yes	Yes †
R-squared	0.022	0.155	0.190	-
Observations	197	197	191	191

Notes: Results from OLS regressions with robust standard errors are reported. Facility and provider characteristics are as shown in Table 1 (type of facility, target population, competition, distance to higher-level facility, facility participation in a results-based financing scheme, proportion of essential drugs and equipment available; provider gender, skill, experience, training). All models control for SP fixed effects. Model 2 – Model 3 control for the number of patients waiting when SPs arrived and for whether providers had been informed of SPs visits beforehand. † Model 4 includes only controls selected via post-double-selection (PDS) lasso (i.e. SP fixed effects, facility type as well as provider skills). *** p<0.01, ** p<0.05, * p<0.1 *** p<0.01, ** p<0.05, * p<0.1

Table A4 – Treatment effects on symptom monitoring advice to patients

	Return if symptoms worsen	Return if blood in sputum	Return if symptoms worsen or blood in sputum
	(1)	(2)	(3)
Patient discloses more information	-0.015	-0.014	-0.007
	(0.051)	(0.019)	(0.052)
Facility characteristics	Yes	Yes	Yes
Provider characteristics	No	No	No
R-squared	0.125	0.045	0.115
Observations	197	197	197

Notes: Results from OLS regressions are reported. All models control for facility and provider characteristics (type of facility, target population, competition, distance to higher-level facility, facility participation in a results-based financing scheme, proportion of essential drugs and equipment available; provider gender, skill, experience, training). *** p<0.01, ** p<0.05, * p<0.1

A.6 Differences in effort in the consultation across treatments

Table A5 – Questions asked by providers

	Treatment (N=100)		Control (N=97)			
	Mean	SD	Mean	SD	diff	p-val
Blood in sputum?	0.41	0.49	0.27	0.45	0.14	0.04
People in surrounding cough?	0.24	0.43	0.10	0.31	0.14	0.01
Weight loss?	0.31	0.46	0.24	0.43	0.07	0.25
Cough or asthma in family?	0.11	0.31	0.04	0.20	0.07	0.07
Chest pain?	0.17	0.38	0.12	0.33	0.05	0.36
Trouble breathing?	0.05	0.22	0.04	0.20	0.01	0.77
Night sweats?	0.18	0.39	0.19	0.39	-0.01	0.92
Loss of appetite?	0.19	0.39	0.22	0.41	-0.03	0.65
Exposed to cigarette smoke?	0.12	0.33	0.15	0.36	-0.03	0.48
Chills?	0.05	0.22	0.09	0.29	-0.04	0.24
Duration of cough?	0.73	0.45	0.79	0.41	-0.06	0.30
Productive cough?	0.43	0.50	0.53	0.50	-0.10	0.18
Cough lasts all day?	0.16	0.37	0.26	0.44	-0.10	0.09
Fever?	0.54	0.50	0.65	0.48	-0.11	0.12
Any other symptoms	0.36	0.48	0.47	0.50	-0.11	0.10
How do you feel in general?	0.13	0.34	0.32	0.47	-0.19	0.00
Weight loss AND blood in sputum	0.18	0.39	0.08	0.28	0.10	0.04
Weight loss OR blood in sputum	0.36	0.48	0.34	0.48	0.02	0.77

Table A6 – Physical examinations done by providers

	Treatment (N=100)		Control (N=97)			
	Mean	SD	Mean	SD	diff	p-val
Take temperature	0.84	0.37	0.84	0.37	0.00	0.93
Take blood pressure	0.77	0.42	0.79	0.41	-0.02	0.69
Take pulse	0.25	0.44	0.24	0.43	0.01	0.83
Take respiratory frequency	0.06	0.24	0.07	0.26	-0.01	0.73
Use stethoscope on front	0.31	0.46	0.32	0.47	-0.01	0.89
Use stethoscope on back	0.27	0.45	0.29	0.46	-0.02	0.77

A.7 Vignette experiment

The 119 healthcare workers who indicated that they conduct curative adult consultations were asked to complete the clinical vignette task. Providers were randomised to assess a hypothetical patient using either the generic introduction (low-information vignette, n=67) or the detailed introduction (high-information vignette, n=52) – based on a random draw. As shown in Table A5 below, we find that the randomisation was successful, as providers in each group are balanced on observed characteristics.

Table A7– Balance checks for vignette experiment

	High-information vignette		Low-information vignette		p-val.
	Mean	SD	Mean	SD	
Panel A: Facility characteristics					
Health post	0.94	0.24	0.93	0.26	*0.72*
Target population size (thousands)	7819	8019	6949	5639	*0.49*
Competition (facilities in 5km radius)	1.75	3.47	1.66	2.45	*0.86*
Distance to next higher-level facility (km)	34.96	34.94	28.76	32.96	*0.32*
% of essential drugs and equipment available	0.80	0.08	0.79	0.08	*0.76*
Treatment guidelines for TB available	0.54	0.50	0.40	0.49	*0.14*
Panel B: Provider characteristics					
Male	0.63	0.49	0.61	0.49	*0.80*
Skilled (nurse, doctor, midwife)	0.48	0.50	0.48	0.50	*0.97*
Work experience (years)	9.13	6.91	11.67	10.20	*0.13*
Undertook training on TB	0.38	0.49	0.27	0.45	*0.18*
Intends to quit job	0.29	0.46	0.25	0.44	*0.67*
N	119		119		

Note: p-values of t-tests for means and chi-squared tests for proportions.

Table A8 – Effect of information on performance in vignettes

	Correct management in vignettes		Effort (IRT score) in vignettes		% questions asked and examinations done in vignettes	
	(1)	(2)	(3)	(4)	(5)	(6)
High-information vignette	-0.049	-0.034	0.054	0.023	-0.011	-0.013
	(0.076)	(0.077)	(0.194)	(0.204)	(0.025)	(0.026)
Mean in low-information vignette	0.84	0.84	0.08	0.08	0.34	0.34
Facility characteristics	Yes	Yes	Yes	Yes	Yes	Yes
Provider characteristics	No	Yes	No	Yes	No	Yes
R-squared	0.077	0.090	0.081	0.104	0.057	0.101
Observations	119	119	119	119	119	119

Notes: Results from OLS regressions with robust standard errors are reported. Facility and provider characteristics are as shown in Table 1 (type of facility, target population, competition, distance to higher-level facility, facility participation in a results-based financing scheme, proportion of essential drugs and equipment available; provider gender, skill, experience, training). The IRT score has a mean of zero and a standard deviation of one. *** p<0.01, ** p<0.05, * p<0.1

A.8 Heterogeneous effects

Table A9 – Heterogeneous treatment effects by provider knowledge

	Correct management		Effort (IRT score)	
	(1)	(2)	(3)	(4)
Patient discloses more information	0.086	0.205**	0.240	-0.045
	(0.177)	(0.078)	(0.437)	(0.121)
High knowledge (in vignette)	-0.171		0.330	
	(0.140)		(0.346)	
Patient discloses more information X high knowledge	0.158		-0.575	
	(0.196)		(0.484)	
Competence index		-0.018		0.447***
		(0.059)		(0.100)
Patient discloses more information X Competence index		-0.025		0.126
		(0.082)		(0.125)
Observations	119	119	119	119

Notes: Results from OLS regressions are reported. All models control for facility and provider characteristics (type of facility, target population, competition, distance to higher-level facility, facility participation in a results-based financing scheme, proportion of essential drugs and equipment available; provider gender, skill, experience, training). Provider knowledge is measured in terms of: whether providers correctly managed an identical case of TB in the vignette task as well as a competence index representing the weighted proportion of clinically indicated history questions asked and examinations performed in the vignette (aggregated using item-response theory) *** p<0.01, ** p<0.05, * p<0.1

Table A10 – Heterogeneous treatment effects on correct management by provider motivation

	Correct management (1)
Patient discloses more information	0.097
	(0.087)
Low motivation	-0.196
	(0.123)
Patient discloses more information X low motivation	0.304*
	(0.171)
R-squared	0.275
Observations	134

Notes: Results from OLS regressions with robust standard errors are reported. Facility and provider characteristics are as shown in Table 1 (type of facility, target population, competition, distance to higher-level facility, facility participation in a results-based financing scheme, proportion of essential drugs and equipment available; provider gender, skill, experience, training). All models control for SP fixed effects. The IRT score has a mean of zero and a standard deviation of one. *** p<0.01, ** p<0.05, * p<0.1.

Table A11 – Heterogeneous treatment effects of effort by provider motivation

	Effort (IRT score)	% questions asked and examinations done	Duration (minutes)
	(1)	(2)	(3)
Patient discloses less information	0.224	0.058**	0.985
	(0.199)	(0.029)	(1.110)
Low motivation	0.122	0.039	-1.022
	(0.273)	(0.039)	(1.524)
Patient shares less information X low motivation	0.177	0.023	-0.523
	(0.391)	(0.056)	(2.179)
R-squared	0.246	0.228	0.390
Observations	134	134	134

Notes: Results from OLS regressions with robust standard errors are reported. Facility and provider characteristics are as shown in Table 1 (type of facility, target population, competition, distance to higher-level facility, facility participation in a results-based financing scheme, proportion of essential drugs and equipment available; provider gender, skill, experience, training). All models control for SP fixed effects. The IRT score has a mean of zero and a standard deviation of one. *** p<0.01, ** p<0.05, * p<0.1.

A.9 Robustness checks

A.9.1 Alternative mechanisms

Table A12 – Differences in providers' bedside manners across treatment groups

	Treatment		Control		p-val.
	Mean	SD	Mean	SD	
Provider was attentive♣	0.87	0.34	0.85	0.36	0.62
Provider cared about my problems♣	0.76	0.43	0.71	0.46	0.44
Provider was not listening to what I was saying♣	0.03	0.17	0.04	0.20	0.67
Provider looked at me when I spoke♣	0.90	0.30	0.87	0.34	0.46
Provider was unkind to me♣	0.03	0.17	0.01	0.10	0.33
Provider explained things well to me♣	0.64	0.48	0.56	0.50	0.24
Provider made me feel uncomfortable♣	0.09	0.29	0.11	0.32	0.55
Provider was on phone during consultation	0.14	0.35	0.07	0.26	0.12
Provider spoke to other patients during consultation	0.09	0.29	0.11	0.32	0.59

Note: ♣ Refers to the proportion of SPs who indicated that they agree of strongly agree with the statement. p-values of t-tests for means and chi-squared tests for proportions.

Table A13 – Treatment effects on correct case management (undetected SPs only)

	Correct case management		
	(1)	(2)	(3)
Patient discloses more information	0.192***	0.198***	0.192***
	(0.069)	(0.070)	(0.070)
Facility characteristics	No	Yes	Yes
Provider characteristics	No	No	Yes
R-squared	0.107	0.166	0.216
Observations	180	180	174

Notes: Results from OLS regressions are reported. The outcome in all models is the probability of correct case management. All models control for SP fixed effects. Facility and provider characteristics are as shown in Table 1 (type of facility, target population, competition, distance to higher-level facility, facility participation in a results-based financing scheme, proportion of essential drugs and equipment available; provider gender, skill, experience, training). Model 2 – Model 3 control for the number of patients waiting when SPs arrived and for whether providers had been informed of SPs visits beforehand. *** p<0.01, ** p<0.05, * p<0.1

A.9.2 Increasing precision of estimates

Table A14 – Treatment effects on quality of care (knowledge controls)

	Correct case management			
	(1)	(2)	(3)	(4)
Patient discloses more information	0.215***	0.160**	0.228***	0.212***
	(0.076)	(0.066)	(0.075)	(0.078)
Correctly managed TB vignette	-0.090			
	(0.097)			
Competence index for TB (IRT)		-0.030		-0.090
		(0.034)		(0.099)
Proportion of vignettes correctly managed			0.465**	
			(0.223)	
Correctly managed dysentery vignette				0.085
				(0.086)
Correctly managed malaria vignette				-0.001
				(0.101)
Correctly managed pneumonia vignette				-0.080
				(0.146)
Correctly managed asthma vignette				0.197**
				(0.093)
Correctly managed angina vignette				0.064
				(0.096)
R-squared	0.310	0.212	0.346	0.361
Observations	119	191	121	118

Notes: Results from OLS regressions with robust standard errors are reported. The outcome in all models is the probability of correct case management. Facility and provider characteristics are as shown in Table 1 (type of facility, target population, competition, distance to higher-level facility, facility participation in a results-based financing scheme, proportion of essential drugs and equipment available; provider gender, skill, experience, training). *** p<0.01, ** p<0.05, * p<0.1

Table A15 – Treatment effects on provider effort

	Provider effort (IRT score)			
	(1)	(2)	(3)	(4)
Patient discloses more information	-0.228	-0.047	-0.224	-0.200
	(0.189)	(0.121)	(0.185)	(0.198)
Correctly managed TB vignette	0.034			0.024
	(0.241)			(0.253)
Competence index for TB (IRT)		0.525***		
		(0.062)		
Proportion of vignettes correctly managed			0.718	
			(0.525)	
Correctly managed dysentery vignette				0.052
				(0.218)
Correctly managed malaria vignette				-0.057
				(0.255)
Correctly managed pneumonia vignette				0.196
				(0.368)
Correctly managed asthma vignette				0.075
				(0.235)
Correctly managed angina vignette				0.317
				(0.242)
R-squared	0.245	0.434	0.259	0.278
Observations	119	191	121	118

Notes: Results from OLS regressions with robust standard errors are reported. The outcome in all models is provider effort (IRT score). Facility and provider characteristics are as shown in Table 1 (type of facility, target population, competition, distance to higher-level facility, facility participation in a results-based financing scheme, proportion of essential drugs and equipment available; provider gender, skill, experience, training). The IRT score has a mean of zero and a standard deviation of one. *** p<0.01, ** p<0.05, * p<0.1

Table A16 – Treatment effects on the duration of the consultation

	Duration (minutes)			
	(1)	(2)	(3)	(4)
Patient discloses more information	-0.569	0.179	-0.516	-0.370
	(1.029)	(0.741)	(1.019)	(1.070)
Correctly managed TB vignette	1.246			1.695
	(1.309)			(1.345)
Competence index for TB (IRT)		0.719*		
		(0.385)		
Proportion of vignettes correctly managed			2.847	
			(2.884)	
Correctly managed dysentery vignette				0.282
				(1.179)
Correctly managed malaria vignette				-1.521
				(1.380)
Correctly managed pneumonia vignette				2.537
				(1.989)
Correctly managed asthma vignette				-0.721
				(1.269)
Correctly managed angina vignette				1.115
				(1.309)
R-squared	0.387	0.331	0.391	0.412
Observations	119	191	121	118

Notes: Results from OLS regressions with robust standard errors are reported. The outcome in all models is the duration of the consultation. Facility and provider characteristics are as shown in Table 1 (type of facility, target population, competition, distance to higher-level facility, facility participation in a results-based financing scheme, proportion of essential drugs and equipment available; provider gender, skill, experience, training). *** p<0.01, ** p<0.05, * p<0.1

Table A17– Treatment effects on correct case management (PDS lasso)

	Correct case management			
	(1)	(2)	(3)	(4)
Patient discloses more information	0.263***	0.157**	0.260***	0.251***
	(0.074)	(0.062)	(0.069)	(0.077)
Correctly managed TB vignette	-0.109			-0.106
	(0.081)			(0.082)
Competence index for TB (IRT)		-0.031		
		(0.033)		
Proportion of vignettes correctly managed			0.239	
			(0.187)	
Correctly managed dysentery vignette				0.025
				(0.068)
Correctly managed malaria vignette				0.072
				(0.080)
Correctly managed pneumonia vignette				-0.129
				(0.126)
Correctly managed asthma vignette				0.096
				(0.073)
Correctly managed angina vignette				0.159*
				(0.096)
Observations	119	191	121	118

Notes: Results from OLS regressions with robust standard errors are reported. The outcome in all models is the probability of correct case management. The outcome in all models is the probability of correct case management. Controls were selected using Post-Double-Selection (PDS) LASSO. Models 1 and 4 include no additional controls. Models 2 and 3 control for whether providers are skilled. *** p<0.01, ** p<0.05, * p<0.1

Table A18 – Treatment effects on the duration of the consultation (PDS lasso)

	Duration (minutes)			
	(1)	(2)	(3)	(4)
Patient discloses more information	-0.892	-0.070	-0.898	-0.617
	(0.979)	(0.699)	(0.971)	(0.946)
Correctly managed TB vignette	1.794*			2.180**
	(1.018)			(1.033)
Competence index for TB (IRT)		0.621*		
		(0.356)		
Proportion of vignettes correctly managed			3.513	
			(2.356)	
Correctly managed dysentery vignette				0.623
				(1.046)
Correctly managed malaria vignette				-1.818*
				(0.990)
Correctly managed pneumonia vignette				2.926**
				(1.299)
Correctly managed asthma vignette				-0.518
				(0.971)
Correctly managed angina vignette				0.771
				(1.367)
Observations	119	191	121	118

Notes: Results from OLS regressions with robust standard errors are reported. The outcome in all models is the duration of the consultation. Controls were selected using Post-Double-Selection (PDS) LASSO. Model 2 includes provider sex. Other models include no controls. *** p<0.01, ** p<0.05, * p<0.1

Table A19 – Treatment effect on provider effort (PDS lasso)

	Effort (IRT score)	% questions asked and examinations done	Duration (minutes)
	(1)	(2)	(3)
Patient discloses more information	0.030	0.026	0.113
	(0.139)	(0.019)	(0.700)
Observations	191	191	191

Notes: Results from OLS regressions with robust standard errors are reported. Models only include controls selected via Post-Double-Selection (PDS) LASSO. Model 1 and 2 include no control variables and Model 3 controls for provider gender. The IRT score has a mean of zero and a standard deviation of one. *** p<0.01, ** p<0.05, * p<0.1

Table A20 – Heterogeneous treatment effects by provider motivation (PDS lasso)

	Correct management	Effort (IRT score)	% questions asked and examinations done	Duration (minutes)
	(1)	(2)	(3)	(4)
Patient discloses less information	-0.130	0.067	0.037	1.375
	(0.085)	(0.179)	(0.025)	(1.054)
Low motivation	0.105	0.191	0.039	-0.994
	(0.083)	(0.283)	(0.040)	(1.458)
Patient shares less information X low motivation	-0.338**	0.247	0.030	-0.671
	(0.165)	(0.389)	(0.057)	(2.029)
Observations	134	134	134	134

Notes: Notes: Results from OLS regressions with robust standard errors are reported. Controls were selected using Post-Double-Selection (PDS) LASSO. Models 1 controls for facility type. Models 2-4 include no additional controls. *** p<0.01, ** p<0.05, * p<0.1

Table A21 – Treatment effects on provider effort (PDS lasso)

	Provider effort (IRT score)			
	(1)	(2)	(3)	(4)
Patient discloses more information	-0.094	0.062	-0.086	-0.105
	(0.191)	(0.117)	(0.187)	(0.199)
Correctly managed TB vignette	-0.118			-0.072
	(0.241)			(0.222)
Competence index for TB (IRT)		0.532***		
		(0.060)		
Proportion of vignettes correctly managed			0.742	
			(0.508)	
Correctly managed dysentery vignette				0.030
				(0.211)
Correctly managed malaria vignette				0.099
				(0.247)
Correctly managed pneumonia vignette				0.282

				(0.356)
Correctly managed asthma vignette				0.010
				(0.210)
Correctly managed angina vignette				0.411*
				(0.233)
Observations	119	191	121	118

Notes: Results from OLS regressions with robust standard errors are reported. The outcome in all models is provider effort (IRT score). Controls were selected using Post-Double-Selection (PDS) LASSO. The PDS LASSO procedure did not select any controls. *** p<0.01, ** p<0.05, * p<0.1

A.9.3 Alternative specifications

Table A22 – Effect of patient attitude on correct case management (firth logistic regressions)

	Correct case management		
	(1)	(2)	(3)
High information	0.812**	0.898***	0.883**
	(0.324)	(0.346)	(0.354)
Facility characteristics	No	Yes	Yes
Provider characteristics	No	No	Yes
Observations	197	197	191

Notes: Models show log-odds after firth logistic regressions. The outcome in all models is the probability of correct case management. All models control for facility and provider characteristics (type of facility, target population, competition, distance to higher-level facility, facility participation in a results-based financing scheme, proportion of essential drugs and equipment available; provider gender, skill, experience, training). All models control for SP fixed effects. Model 2 – Model 3 control for the number of patients waiting when SPs arrived and for whether providers had been informed of SPs visits beforehand. *** p<0.01, ** p<0.05, * p<0.1.

Table A23 – Effect of patient attitude on correct case management (probit regressions)

	Correct case management		
	(1)	(2)	(3)
Patient discloses more information	0.162**	0.181***	0.170***
	(0.066)	(0.062)	(0.062)
Facility characteristics	No	Yes	Yes
Provider characteristics	No	No	Yes
Proportion of correct predictions	68.020	75.130	72.590
Pseudo R-squared	0.024	0.161	0.194
Observations	197	197	191

Notes: Marginal effects from probit regressions are reported. The outcome in all models is the probability of correct case management. Facility and provider characteristics are as shown in Table 1 (type of facility, target population, competition, distance to higher-level facility, facility participation in a results-based financing scheme, proportion of essential drugs and equipment available; provider gender, skill, experience, training). All models control for SP fixed effects. Model 2 – Model 3 control for the number of patients waiting when SPs arrived and for whether providers had been informed of SPs visits beforehand. *** p<0.01, ** p<0.05, * p<0.1
